# Intravenous thrombolysis in patient with vertebrobasilar dolichoectasia and antiplatelet medication

**DOI:** 10.1016/j.radcr.2022.06.061

**Published:** 2022-07-18

**Authors:** Pipit Mei Sari, Achmad Firdaus Sani, Dedy Kurniawan

**Affiliations:** Neurology Department, Faculty of Medicine, Universitas Airlangga, Dr. Soetomo General Hospital, Surabaya, Indonesia

**Keywords:** Antiplatelet, Intravenous thrombolysis, Stroke, Vertebrobasilar dolichoectasia, Case report

## Abstract

*Introduction:* While the overall incidence of vertebrobasilar dolichoectasia (VBD) is less than 0.05%-0.06%, it is not uncommon in patients experiencing acute stroke. The influence of VBD on the outcome of intravenous (IV) thrombolysis therapy has not been widely studied. We present the following case of IV thrombolysis use in a patient experiencing acute stroke, who had an increased risk of bleeding due to prior antiplatelet use, and who had concomitant VBD. *Case presentation:* A 62-year-old man presented with weakness in the left extremities that had begun 1 hour prior to admission. The patient had a history of coronary artery disease and had been regularly taking antiplatelet medication. Upon arrival, the patient was in a decreased level of consciousness, with severe dysarthria, left central facial palsy, left lateralization, and a National Institute of Health Stroke Scale (NIHSS) score of 17. Computed tomography scan of the head showed no intracranial hemorrhage. The patient was administered IV thrombolysis at 2 hours and 45 minutes after symptom onset. Within the first 24 hours, the patient's NIHSS score decreased from 17 to 12, and the final NIHSS score prior to discharge was 7. The Head and neck angiography of this patient revealed VBD. *Conclusion*: This case demonstrated that IV thrombolysis is safe and effective for use in patients with acute ischemic stroke who have a history of antiplatelet usage and who experience concomitant VBD.

## Introduction

Intravenous (IV) thrombolysis is a standard recanalization therapy for the treatment of acute ischemic stroke, to be administered within the first 4.5 hours of stroke symptom onset. However, further studies on the safety and efficacy of IV thrombolysis are still needed. Prior antiplatelet usage was not considered exclusion criteria for IV thrombolysis therapy, despite the associated increased risk of a bleeding complication [1]. Vertebrobasilar dolichoectasia (VBD) is an uncommon disease that is characterized by expansion, elongation, and tortuosity of the vertebrobasilar arteries [2], [3], [4]. VBD is not uncommonly found in patients with acute stroke [5], and its unclear pathophysiology may have an impact on the effect of IV thrombolysis. Currently, the outcome of IV thrombolysis in this patient population has not been well documented. The following is a case of IV thrombolysis use in a patient with acute stroke with a history of antiplatelet usage and concomitant VBD.

## Case report

**Fig. 1:**
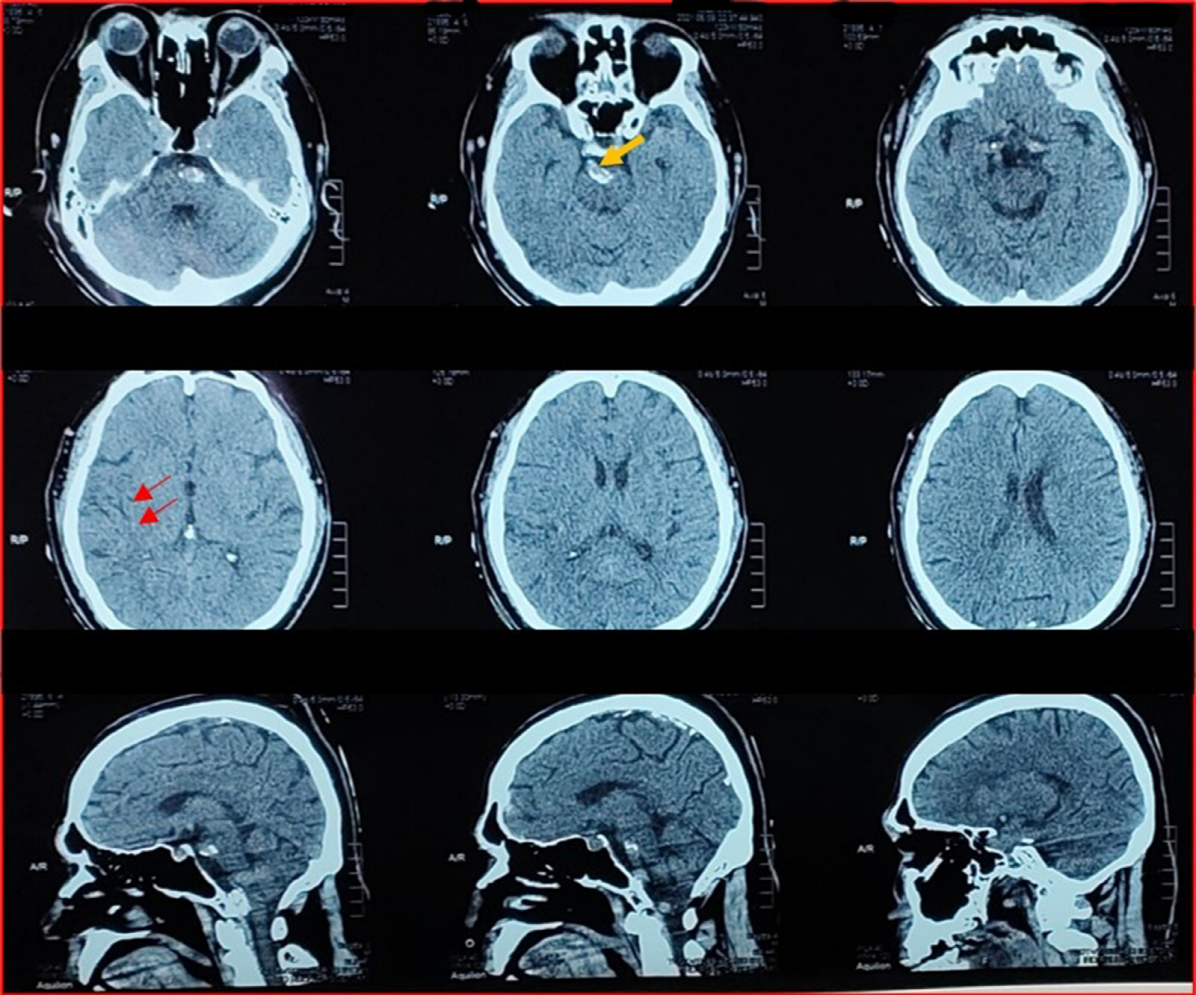
CT scan showed a blurred insular ribbon on the right side (red arrow). The ventral calcification of the pons is shown (orange arrow). (Color version of figure are available online)

A 62-year-old man presented with weakness in the left extremities that had started 1 hour prior to admission. The patient had a history of coronary artery disease and stent placement 2 years earlier. Since then, he has taken antiplatelet medication with aspirin (100 mg) daily. Upon arrival, the patient was screened for COVID-19 via antigen nasopharyngeal swab, and the result was negative. The patient had not received any COVID-19 vaccination, citing multiple comorbidities. His blood pressure was 200/110 mm Hg, and an electrocardiogram showed sinus rhythm with a heart rate of 94 beats per minute, and evidence of an old inferior myocardial infarction. He had a Glasgow Coma Scale of E3V4M5, severe dysarthria, left central facial palsy, and left hemiplegia impression with a National Institute of Health Stroke Scale (NIHSS score of 17). Computed tomography (CT) scan of the head (performed at 1 hour and 45 minutes) revealed the presence of calcifications on the ventral part of the pons, without evidence of intracranial hemorrhage (Fig. 1).

**Fig. 2:**
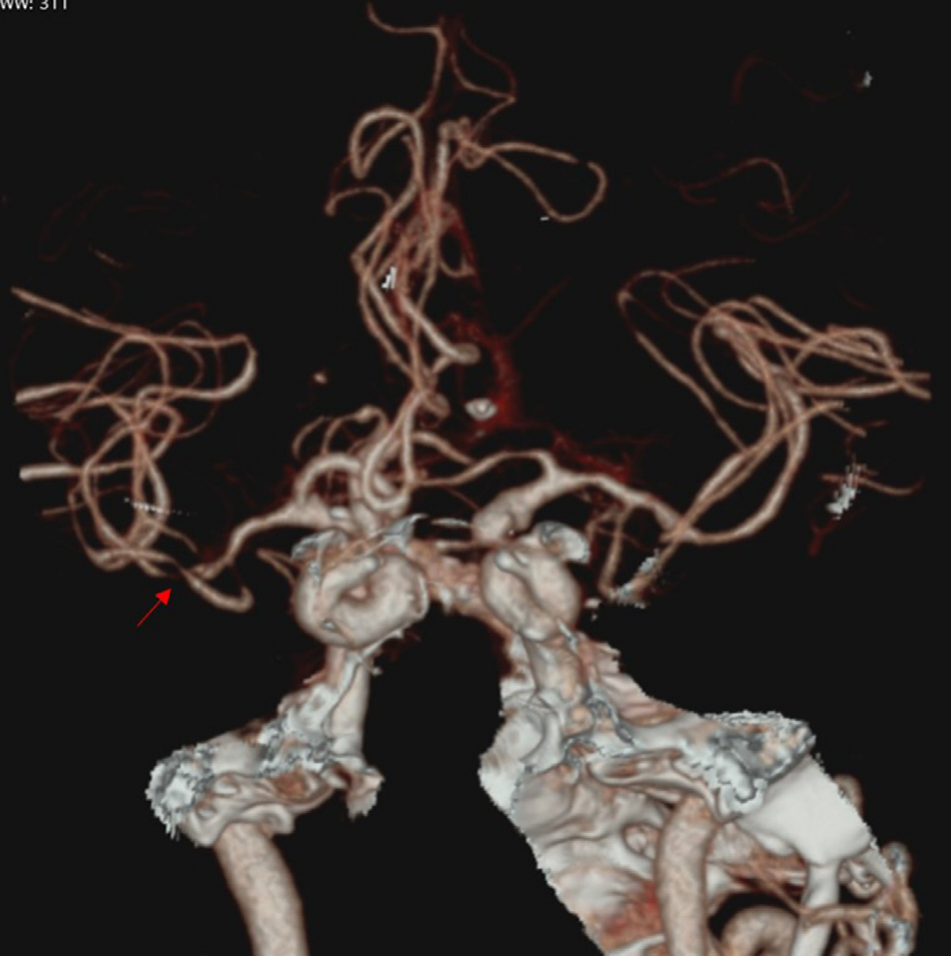
CT Angiography showed arterial patency with stenosis of the right M1-M2 segment (red arrow). (Color version of figure are available online.)

At 2 hours and 45 minutes postsymptom onset, the patient's blood pressure had decreased, and IV thrombolysis was administered. The IV thrombolysis used was alteplase, 72 mg (0.9 mg/kg BW) given over 60 minutes. During administration, no allergic reactions, angioedema, or signs of bleeding were noted. CT Angiography (CTA) acquired one hour after thrombolysis showed stenosis in the M1-M2 region of the right middle cerebral artery (Fig. 2).

**Fig. 3:**
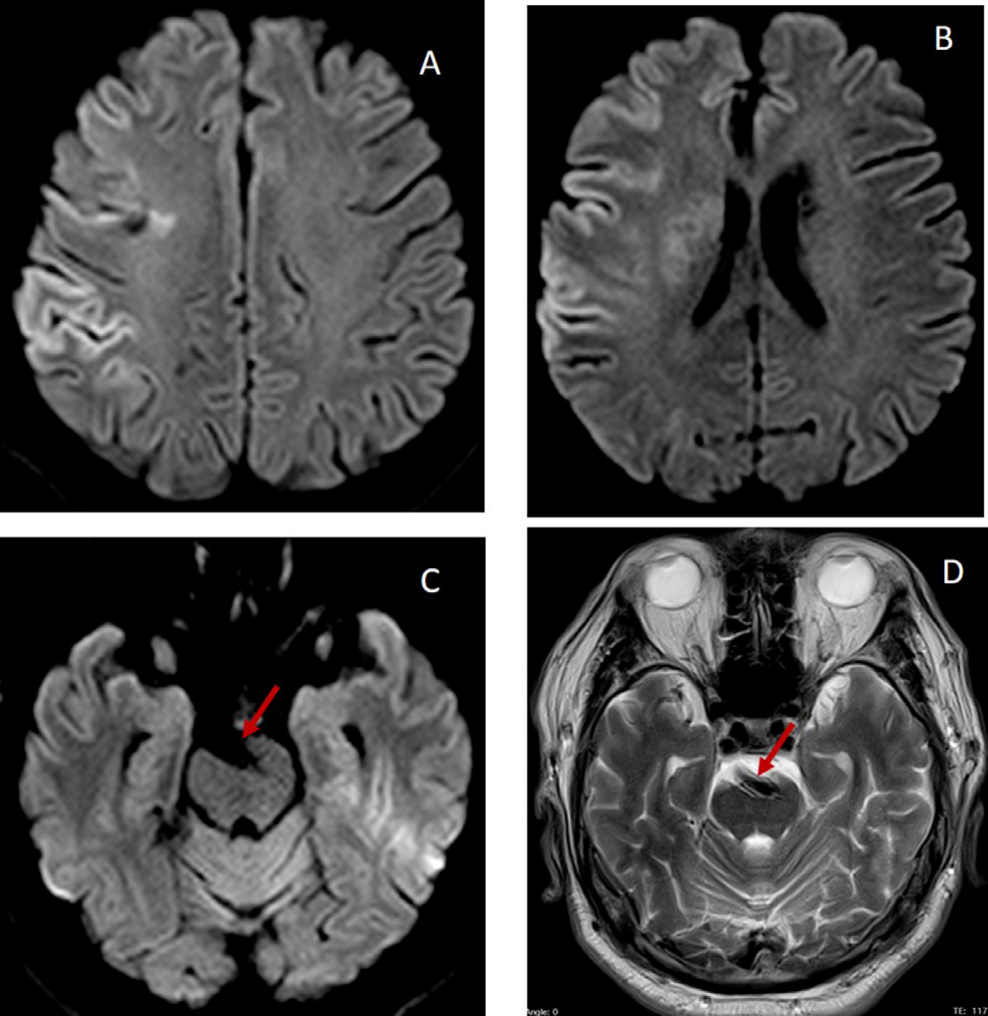
(A and B) Diffusion-weighted imaging (DWI) show an acute right frontal stroke. (C) DWI and (D) T2 show arterial compression on the pons (red arrow). (Color version of figure is available online.)

**Fig. 4:**
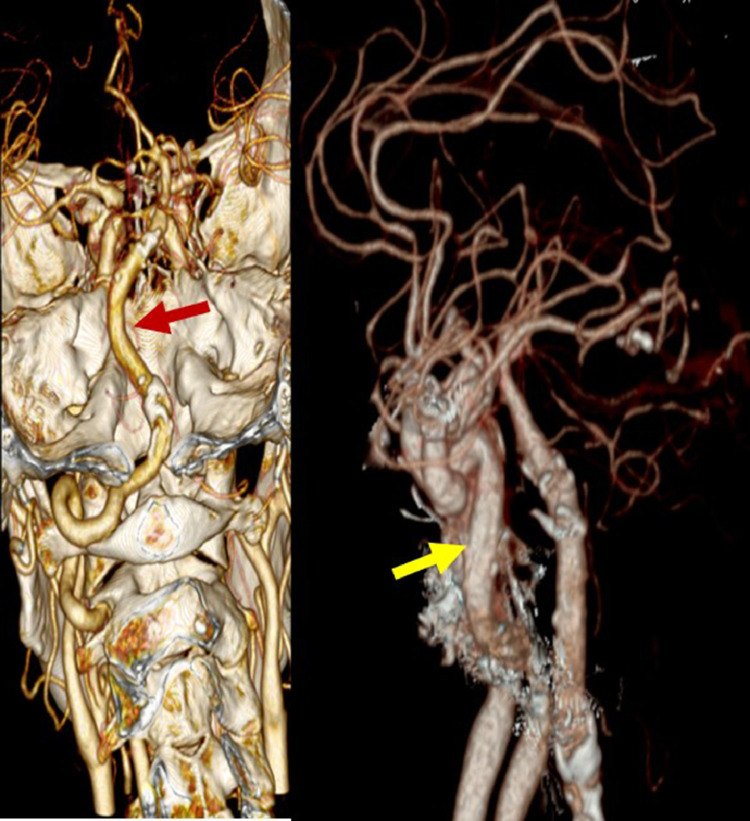
A large vertebrobasilar artery (red arrow) is seen on CT angiography with the same size as the internal carotid artery (yellow arrow). (Color version of figure is available online.)

By the end of the first day, the patient's NIHSS score had decreased from 17 to 12. Magnetic resonance imaging of the head performed on the sixth day of admission showed an acute ischemic stroke in the right frontal region, and arterial compression on the pons (Fig. 3). Vertebrobasilar artery dilatation and tortuosity were also seen with the same size as the internal carotid artery (Fig. 4), suggesting VBD. On the seventh (final) day of admission, the patient's residual deficits included mild dysarthria, left facial palsy, and left hemiparesis (left limb motoric score of 4), with an NIHSS score of 7.

## Discussion

Stroke is the world's second greatest cause of mortality [6], [7], [8], and is a major contributor to the cause of death in developing countries [9,10]. Approximately 25% of newly reported ischemic strokes occur in people with a history of prior stroke [11]. Almost 40% of these patients had used antiplatelet medication as secondary stroke prevention, and therefore this population may be at a higher risk for bleeding complications during the administration of alteplase [12], [13], [14], [15], [16]. In patients treated with antiplatelets, the percentage of spontaneous intracerebral hemorrhage (sICH) after thrombolysis was 9% [17]. Despite this, antiplatelet use was not associated with poorer functional outcomes in patients, and therefore, IV thrombolysis is still recommended as treatment for acute ischemic stroke [18].

The prevalence of VBD was previously estimated at 0.2%-4.4% in the general population, however, angiography and autopsy results indicate that the overall incidence is actually not more than 0.05%-0.06% [2,19,20]. Currently, the mechanisms that contribute to VBD have not been elucidated. Several hypotheses exist, such as depletion of trophic support, reticular fiber deficiency, degeneration of the internal elastic lamina, and smooth muscle atrophy [21,22]. The risk factors associated with VBD include advanced age, hypertension, male sex, and coronary artery disease [23,24], all of which are in line with our patient's history.

Previous research has stated that dysregulation of crosstalk among vascular smooth muscle cells, matrix metalloproteinase, and elastic fiber systems may be involved in VBD [23]. This unexplored mechanism may have a beneficial or harmful effect on IV thrombolysis, but its study was still limited. Chen et al [3] studied seven patients with VBD who received IV thrombolysis, and found that most of them had no recurrence of ischemic stroke. These results may support the beneficial effects of IV thrombolysis use in patients experiencing VBD, but the effect on VBD in patients with prior antiplatelet use has not been reported.

Our patient presented with an acute anterior circulation ischemic stroke, a history of antiplatelet usage, and VBD. Despite the uncertainty of the possible impact of VBD and prior antiplatelet usage on IV thrombolysis, the therapy produced an effective response in this patient. As explained by Gocmen et al, “effective response” was defined as a reduction of the patient's NIHSS score to 1 or 0, or a decrease by 4 points or more in the NIHSS score by the end of the first 24 hours. A “dramatically good response” was defined as a decrease of 8 or more points in the NIHSS score by the end of the first day [23]. Our patient's NIHSS score decreased from 17 to 12 (5 point decrease) by the end of the first 24 hours, defined as an “effective response,” and this might indicate the possibility of beneficial outcomes of IV thrombolysis in this subset of the stroke population.

## Conclusion

VBD is a rare condition, but it is not uncommon in acute stroke patients. VBD coupled with antiplatelet usage may influence the outcome of IV thrombolysis therapy. The increased risk of sICH associated with prior antiplatelet use should not be a reason exclude IV thrombolysis as a treatment for acute ischemic stroke. Despite his underlying conditions, the patient presented in this case study showed significantly improved clinical outcomes at the end of treatment. This case demonstrates that IV thrombolysis may be a safe and effective treatment for patients experiencing acute ischemic stroke who have a history of antiplatelet use and concomitant VBD, although further studies with larger population sizes are warranted.

## Funding

The authors received no financial support for the research, authorship, and/or publication of this article.

## Authors' contributions

PMS and DK contributed to the concept and design of the article, acquisition of patient's data, and drafting of the article. AFS were involved in revising the article critically for important intellectual content.

## Availability of data and material

Data sharing does not apply to this article as no datasets were generated or analyzed during the current study.
